# Supplementary material for: Human Umbilical Mesenchymal Stem Cell Xenografts Repair UV-Induced Photokeratitis in a Rat Model
Source: Biomedicines. 2022 May 12;10(5):1125. doi: 10.3390/biomedicines10051125 (PMC9138504; doi:10.3390/biomedicines10051125)
Supplement: Supplementary file 1 [file biomedicines-10-01125-s001.zip › biomedicines-1706646-supplementary.pdf]

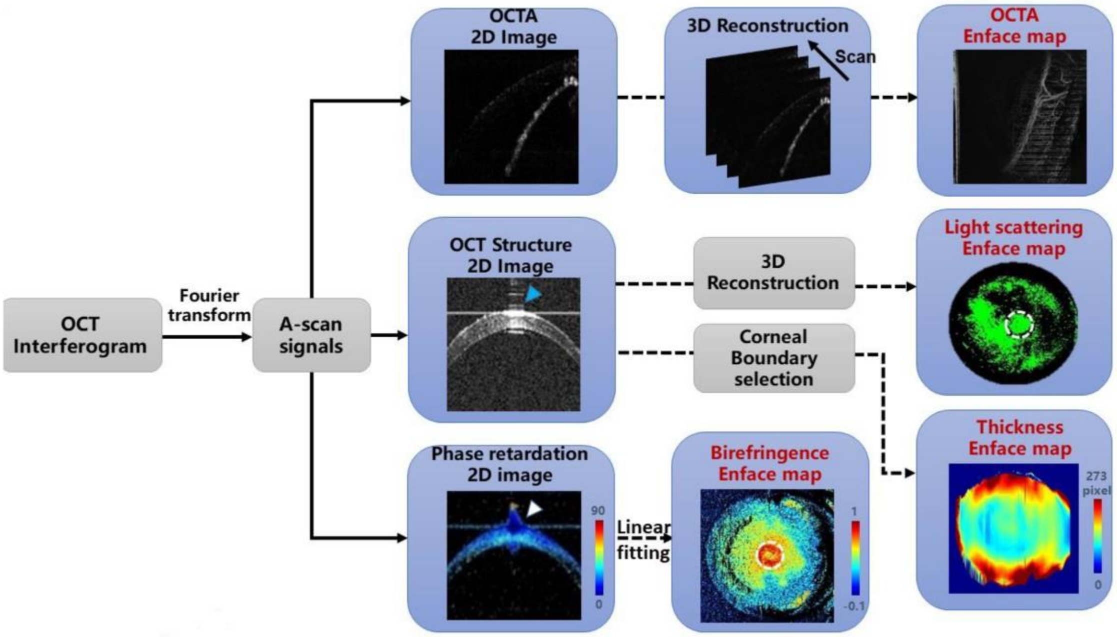

Figure S1. Image processing flowchart.

First, each A-scan signal (i.e., OCT amplitude versus depth) was obtained through the inverse Fourier transformation of the acquired Interferogram. Then, the two- dimensional (2D) cross-sectional structural images (displayed on a logarithmic grayscale) and phase retardation images (presented in color with a range from 0 to 90 degrees) were calculated by using the amplitude of the A-scan signal [1]. Vascular images were acquired through complex signal calculations using an optical microangiography (OMAG) algorithm [2]. A 3D reconstructed data set included a 7 mm × 7 mm × 6 mm volume, corresponding to 1000 × 1000 × 1024 pixels in the x, y, and z directions. Finally, OCTA, light scattering, cornea thickness, and birefringence are shown in a 2D enface (x–y) plane.

Light scattering enface map: Using the intensity value in the OCT structure map on the initial day as the standard, the pixel value was represented with a green color beyond this initial time point. Arrowheads point to the artifacts from highly back-scattered light in the cornea center, represented as a circular region in the enface map and is represented as a black area in Figure 4A. The total pixel number with green color is defined as the light scattering region.

Thickness enface map: The top and lower boundary of the cornea were determined using the same method described in our recently published study [3], and the distance within these two layers was defined as the thickness of the cornea. The color represents the thickness value in the pixel number (1 pixel = 5.85 mm).

Birefringence enface map: For the quantitative analysis of cumulated phase retardation, the birefringence coefficient of each A-line was calculated by linear fitting through the averaged data of four nearby A-lines the slope was determined to construct a birefringence map [4]. Arrowheads point to the artifacts from highly back-scattered light in the cornea center, resulting in a retardation error circled in a red region of the birefringence enface map and covered with a black mask in Figure 4A–C.

OCTA enface map: in order to observe cornea neo-angiogenesis, the eye's side position was used for OCT scanning so that the conjunctiva and cornea could be included in the same field of view simultaneously. The vascular structure visualization in the enface plane was obtained through maximum value projections from the 3D OCTA reconstruction data. As shown in Figure 6A, the arrows mark the large blood vessels at the border of the conjunctiva, whereas the corneal area is on the left side of the large blood vessel. The vessel area in the projection view divided by the imaging region area is defined as the vessel density.

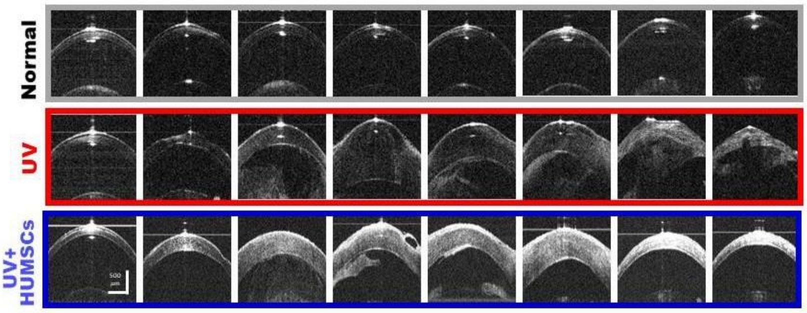

Figure S2. Enface thickness map and representative 2D OCT structure images.

Multi-function OCT shows the enface thickness map (upper part) in which dashed lines illustrate representative 2D OCT structure images (lower part).

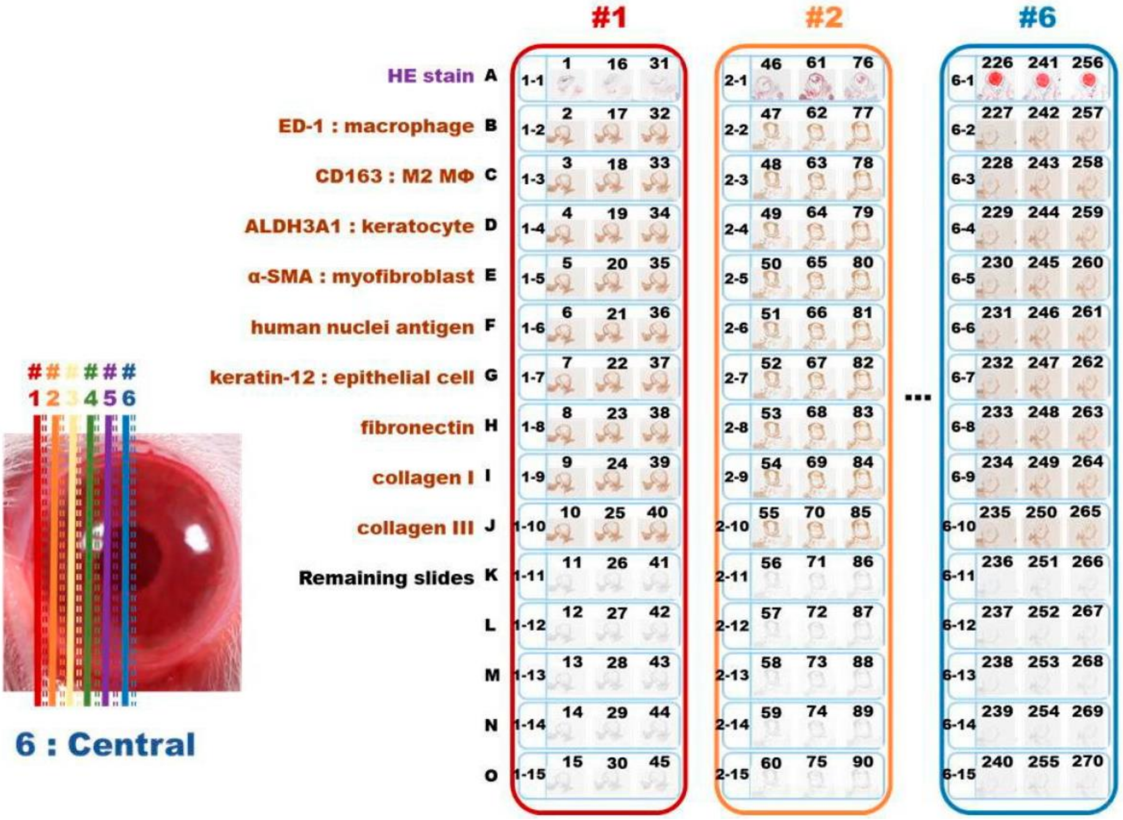

Figure S3. Corneal sectioning methodology for morphology and pathology analysis.

To objectively analyze the pathology of every region of the cornea, they were serially sectioned into 5-μm slices. For example, in the Normal group, lung sagittal slices were numbered from 1 to 15 and placed on slides lettered from A to O. As the sectioning process was performed toward the central region, slices numbered from 16 to 30 and from 31 to 45 were also placed on slides lettered A to J. The procedure was repeated until half of the cornea was completely sectioned. Column #1 represents the outermost region of the cornea, and column #6 represents the central region of the cornea. Thus, corneal slices in row A (numbers: 1, 16, 31, 46, 61, 76, ……, 226, 241, 256) were all subjected to hematoxylin and eosin (H&E) staining, whereas corneal slices in row B (numbers: 2, 17, 32, 47, 62, 77,……226, 242, 257) were subjected to IHC with anti- ED1 antibody to label macrophages. Corneal slices in row C (numbers: 3, 18, 33, 48, 63, 78,……227, 243, 258) were subjected to IHC with anti-CD163 antibody to label M2 macrophages; corneal slices in row D (numbers: 4, 19, 34, 49, 64, 79,……228, 244, 259) were subjected to IHC with anti-ALDH3A1 antibody to label keratocytes; corneal slices in row E (numbers: 5, 20, 35, 50, 65, 80,……229, 245, 260) were subjected to IHC with anti-α-SMA antibody for labeling vessels; corneal slices in row F (numbers: 6, 21, 36, 51, 66, 81,……230, 246, 261) were subjected

to IHC with the anti-human specific nuclear antibody for labeling of HUMSCs; corneal slices in row G (numbers: 7, 22, 37, 52, 67, 82,……231, 247, 262) were subjected to IHC with anti-karatin-12 for labeling epithelial cells; corneal slices in row H (numbers: 8, 23, 38, 53, 68, 83,……232, 248, 263) were subjected to IHC with anti-fibronectin for labeling fibronectin; corneal slices in row I (numbers: 9, 24, 39, 54, 69, 84,……233, 249, 264) were subjected to IHC with anti-collagen I for labeling collagen I; corneal slices in row I (numbers: 10, 25, 40, 55, 70, 85,……234, 250, 265) were subjected to IHC with anti-collagen III for labeling collagen III. The remaining rows (K-O) were preserved as spares.

References

1. Kuo, W.C.; Chou, N.K.; Chou, C.; Lai, C.M.; Huang, H.J.; Wang, S.S.; Shyu, J.J. Polarization-sensitive optical coherence tomography for imaging human atherosclerosis. *Appl. Opt.* **2007**, *46*, 2520–2527.
2. Chen. C.L.; Wang, R.K. Optical coherence tomography-based angiography. *Biomed. Opt. Express* **2017**, *8*, 1056–1082.
3. Lai, P.Y.; Chang, C.H.; Su, H.R.; Kuo, W.C. Lymphatic vessel segmentation in optical coherence tomography by adding U-Net-based CNN for artifact minimization. *Biomed. Opt. Express* **2020**, *11*, 2679–2693.
4. Chen, P.H.; Lee, H.Y.; Chen, Y.F.; Yeh, Y.C.; Chang, K.W.; Hou, M.C.; Kuo, W.C. Detection of oral dysplastic and early cancerous lesions by polarization-sensitive optical coherence tomography. *Cancers* **2020**, *12*, 2376.
